# Clock-Face Sonography of the Glenoid Labrum: A Pictorial Technical Protocol for Patients Ineligible for MRI/MR Arthrography

**DOI:** 10.3390/diagnostics15233031

**Published:** 2025-11-28

**Authors:** Tomasz Poboży, Wojciech Konarski, Kacper Janowski, Klaudia Michalak, Kamil Poboży, Julia Domańska-Poboża, Maciej Kielar

**Affiliations:** 1 Department of Orthopedic Surgery, Medicover Hospital, Aleja Rzeczypospolitej 5, 02-972 Warsaw, Poland; tomasz.pobozy@onet.pl; 2 Medical Rehabilitation Center, Sobieskiego 47D, 05-120 Legionowo, Poland; wkonarski@poczta.onet.pl; 3 Department of Internal Medicine, Specialist Regional Hospital, 06-400 Ciechanow, Poland; kld.michalak@gmail.com; 4 Department of Neurosurgery, Brodnowski Masovian Hospital, 03-242 Warsaw, Poland; pobozykamil@gmail.com; 5 Department of Rheumatology, National Institute of Geriatrics, Rheumatology and Rehabilitation, 02-637 Warsaw, Poland; julia-domanska03@wp.pl; 6 Faculty of Medicine, Lazarski University, 02-662 Warsaw, Poland

**Keywords:** ultrasonography, shoulder, glenoid labrum, clock-face protocol, dynamic imaging, musculoskeletal, MR arthrography contraindications

## Abstract

This work presents a standardized 360-degree, clock-face ultrasonographic protocol for comprehensive static and dynamic assessment of the glenoid labrum. The protocol translates the arthroscopic clock-face orientation into ultrasound scanning windows, providing reproducible steps for each labral quadrant (12 to 12 o’clock) including patient positioning, transducer orientation, and dynamic maneuvers. By leveraging linear transducers with trapezoidal imaging and an optional convex transducer to bypass acoustic shadowing from the acromion and coracoid, all labral segments can be consistently visualized, while dynamic testing reveals subtle clefts, irregular margins, and medial displacement patterns. Clinically, this approach is particularly valuable for patients who cannot undergo MRI or MR arthrography (e.g., due to metallic implants, contrast allergy, claustrophobia or renal dysfunction) and in settings where MR/MRA is unavailable or impractical (sports medicine, urgent care, postoperative follow-up). The pictorial atlas and step-by-step checklists aim to support adoption in routine practice and to facilitate communication with surgeons through shared clock-face terminology. This protocol is not intended to replace MR arthrography for surgical planning; rather, when MRI/MRA cannot be performed or access is limited, it provides actionable, dynamic information that complements clinical decision-making.

## 1. Introduction

The glenoid labrum is a fibrocartilaginous ring that surrounds the margin of the glenoid cavity, deepening its concavity and enhancing the stability of the glenohumeral joint. The labrum is not uniform in its morphology: the superior labrum merges with the tendon of the long head of the biceps brachii, and both structures attach conjointly to the supraglenoid tubercle. Beneath the superior labrum, a sublabral recess may be present—a normal variant that can mimic a tear on imaging. Similarly, an anterosuperior sublabral foramen may occur as an anatomical variant, representing a small gap between the labrum and the glenoid rim. The glenohumeral ligaments, which overlie the anterior portion of the joint capsule, reinforce this region and provide primary static stabilization against anterior translation, while the labrum itself lies deeper to these ligaments along the anterior glenoid rim. In contrast, the inferior and posterior labrum lie directly beneath the joint capsule, which in these regions is thinner and not reinforced by ligaments. This anatomical diversity contributes to variations in both normal sonographic appearance and pathological presentations.

Labral injuries are common in athletes and individuals with recurrent shoulder instability. The Bankart lesion, typically involving detachment of the anteroinferior labrum, often results from anterior shoulder dislocation and may be accompanied by a bony Bankart lesion—an avulsion fracture of the anteroinferior glenoid rim. This may manifest as either subtle rounding of the glenoid contour or a clearly displaced bony fragment. Conversely, the SLAP (superior labrum anterior to posterior) tear affects the superior labrum and the biceps anchor complex, compromising the stability of the biceps–labral junction. Although posterior labral tears are less frequent, they occur in athletes exposed to repetitive posterior shoulder loading, such as boxers, weightlifters, and football linemen, and may lead to subtle posterior instability that often remains clinically underdiagnosed.

Magnetic resonance (MR) arthrography remains the reference standard for the assessment of the glenoid labrum due to its high soft-tissue contrast and excellent visualization of intra-articular anatomy. However, it is invasive, expensive, and time-consuming, and may be contraindicated in patients with contrast allergy, renal dysfunction, selected metallic implants, or claustrophobia. Magnetic resonance imaging (MRI) without intra-articular contrast also has limitations in detecting small or partial labral tears, particularly in the anterosuperior quadrant.

In contrast, ultrasound (US) offers a safe, non-invasive, and dynamic imaging alternative. It allows real-time assessment of soft tissues and their motion, providing functional information beyond static morphology. Although traditionally considered limited to evaluation of the posterior labrum, advances in high-frequency transducers, trapezoidal and convex imaging modes, and improved beam steering have significantly expanded its diagnostic reach. Building upon these technical developments and adopting the arthroscopic clock-face model as an anatomical reference framework, we developed a systematic ultrasonographic protocol for comprehensive 360-degree visualization of the glenoid labrum. The proposed technique defines reproducible scanning windows for each labral segment and incorporates dynamic maneuvers to reveal subtle clefts, marginal irregularities, and displacement patterns [1,2,3,4,5,6,7,8,9].

This standardized approach aims to enhance reproducibility, facilitate communication between sonographers and surgeons, and provide a practical diagnostic option for patients who are ineligible for MRI or MR arthrography or when these modalities are unavailable in clinical practice.

## 2. Materials and Methods

### 2.1. Study Design and Ethical Considerations

This work represents a prospective technical development and feasibility study aimed at establishing a standardized ultrasonographic protocol for comprehensive 360-degree assessment of the glenoid labrum. The protocol was developed and refined through examinations of 36 shoulders, including both asymptomatic volunteers and patients referred for shoulder ultrasonography due to instability symptoms or pain. All examinations were performed by two musculoskeletal sonographers with over ten years of experience, using high-frequency linear transducers and trapezoidal or convex modes as indicated.

The study involved a non-invasive diagnostic procedure (ultrasound) and did not include any therapeutic interventions, randomization, or collection of identifiable personal or clinical data. In accordance with local regulations governing non-interventional imaging research, formal approval from a bioethics committee was not required.

All participants whose images were used for educational or illustrative purposes provided written informed consent for image acquisition and anonymized publication. For representative pathological cases, patients also signed consent for the use of their de-identified clinical images for scientific dissemination. The study was conducted in full compliance with the ethical standards of the Declaration of Helsinki.

### 2.2. Scanning Protocol (Tutorial)

#### 2.2.1. Equipment

- Linear transducer: 3–12 MHz, trapezoidal mode available.
- Convex transducer: 1–6 MHz for deeper penetration and bypassing bony shadowing.

#### 2.2.2. Patient Positioning

- Superior and anterior labrum (12–4 o’clock): Patient seated, arm neutral or externally rotated. In obese patients, a supine position with the arm externally rotated at the 3–4 o’clock position may be better.
- Inferior labrum (5–7 o’clock): supine; maximal abduction and external rotation; examiner on the axillary side.
- Posterior labrum (8–11 o’clock): seated; transducer aligned with the scapular axis; begin at 9 o’clock.

### 2.3. Clock-Face Windows and Maneuvers

#### 2.3.1. Anterosuperior Segment (1–2 O’clock)

The anterosuperior labrum is examined using an oblique anterosuperior approach, with the focal depth adjusted to include both the glenoid rim and the adjacent humeral head. When the acoustic window is limited by the coracoid process or the anterior acromial edge, trapezoidal mode or a convex transducer should be activated. Positioning the labrum near the lateral edge of the field helps to optimize beam incidence and minimize anisotropy.

#### 2.3.2. Anterior Segment (3–4 O’clock)

For visualization of the anterior labrum, the transducer is placed horizontally, with the humeral head located laterally and the glenoid rim medially. External rotation of the arm and gentle medial pressure with the transducer help correct scapular tilt and improve acoustic contact. During dynamic assessment, internal rotation may demonstrate subtle medial displacement of the labral edge, which is a useful indicator of early detachment.

#### 2.3.3. Inferior Segment (5–7 O’clock)

The inferior labrum is best evaluated in the supine position, with the shoulder maximally abducted and externally rotated. Scanning should begin at the 6 o’clock position, followed by approximately 30° anterior or posterior rotation of the transducer to display the 5 and 7 o’clock regions. In this portion of the joint, the capsule is thin and not reinforced by glenohumeral ligaments, which makes assessment relatively straightforward but also requires delicate transducer handling to avoid compression artifacts.

#### 2.3.4. Posterior Segment (8–10 O’clock)

The posterior labrum is assessed with the patient seated and the transducer aligned along the long axis of the scapula. The 9 o’clock position typically provides the clearest window, while slight cranial or caudal rotation of the transducer allows visualization of the 8 and 10 o’clock positions. Maintaining consistent transducer pressure and alignment with the scapular plane ensures stable imaging of this region.

#### 2.3.5. Posterosuperior and Superior Segment (11–12 O’clock)

Acoustic shadowing from the posterior acromial angle may partially obscure the posterosuperior labrum. To overcome this limitation, the transducer should be positioned so that the acromial corner appears at one edge of the image and the glenoid rim at the other. When necessary, trapezoidal imaging mode or a convex transducer can be used to extend the field of view and “peek” beneath the acromion, providing optimal visualization of the superior labral attachment.

### 2.4. Image Atlas and Pitfalls

#### General Figure Legend

**Figure 1:**
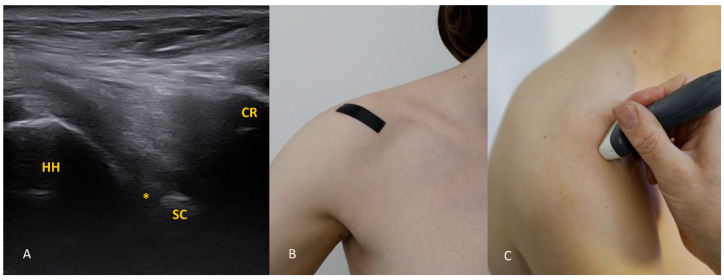
The 1 o’clock position. Imaging at this position is generally not difficult. Bear in mind that the structures of interest (the labrum and the bony glenoid rim) lie relatively deep, so the focal depth should be set appropriately and, if needed, the transducer frequency reduced. The labrum should be sought in the central part of the field.

**Figure 2:**
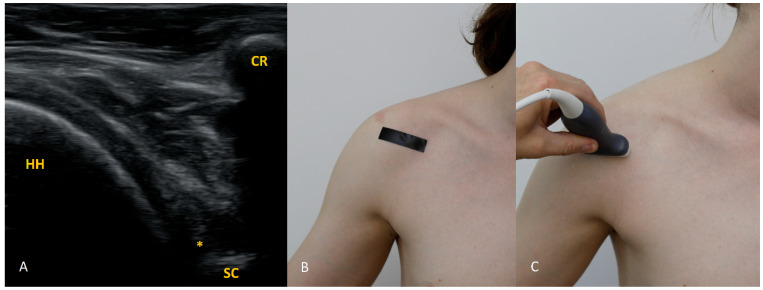
The 2 o’clock position. At 2 o’clock, if the expected location of the labrum and glenoid rim is obscured by acoustic shadow from the coracoid process, use trapezoidal imaging and attempt to display the labrum near one edge of the field. Remember that with trapezoidal imaging, ultrasound beams emitted from the central part of the transducer propagate perpendicularly to the transducer face, whereas those near the transducer ends travel obliquely (with the angle increasing toward the transducer edge). Exploiting these oblique beams allows you to “see” partially beneath shadowing structures. The same effect can be achieved with a convex transducer.

**Figure 3:**
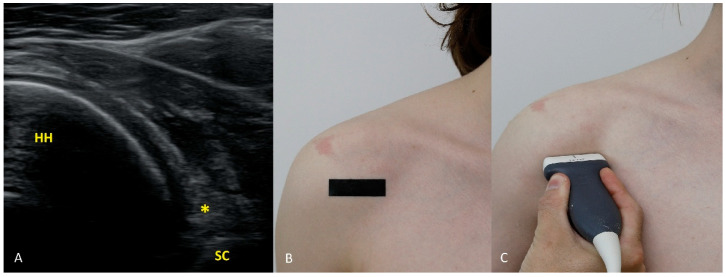
The 3 o’clock position. Typical transducer position and labral appearance. At 3 o’clock, if the patient has substantial muscle mass or is obese, the labrum may be examined with the patient supine. In addition to external rotation and firmer pressure on the medial transducer edge, the supine position helps compensate for the oblique orientation of the scapula relative to the chest wall. The anterior and anteroinferior labrum are the most frequent sites of injury. When looking for signs of damage, pay attention to the labral contour, the presence of fissures that may appear only during dynamic assessment (with rotation), and any medial displacement, which may be fixed (in chronic lesions) or occur only during internal rotation. In that case, during internal rotation the labrum slides medially off the glenoid rim, whereas with external rotation it returns to its proper position (reduction) under tension from the glenohumeral ligaments [8,9,10].

**Figure 4:**
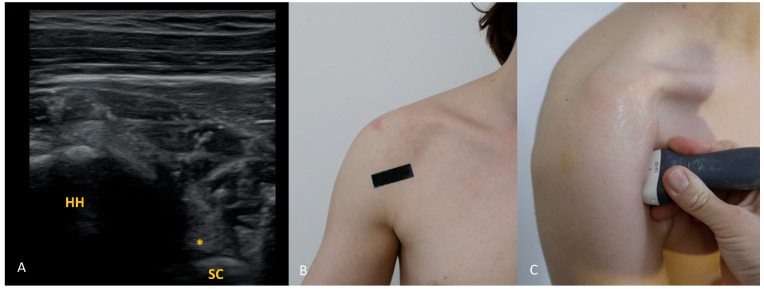
The 4 o’clock position. By moving the transducer slightly inferiorly and tilting it about 30° caudally, the labrum at 4 o’clock is visualized. In addition to external rotation, we recommend abducting the arm by approximately 30°.

**Figure 5:**
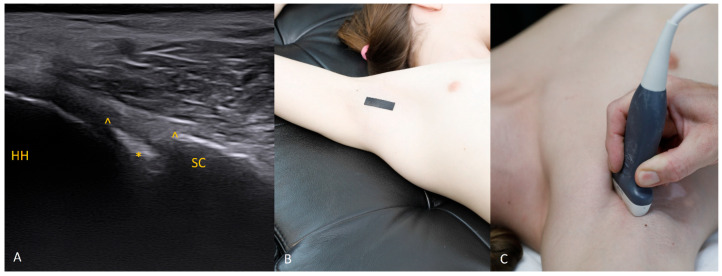
The 5 o’clock position. Relative to imaging at 6 o’clock (Figure 6), the transducer is rotated approximately 30° anteriorly; the scapular body disappears from the image.

**Figure 6:**
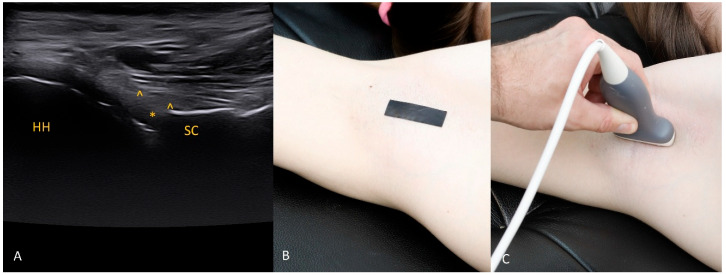
The 6 o’clock position. The transducer is positioned so that the humeral head is seen on one side, the glenoid on the other, and a narrow portion of the scapular body is visible, confirming the exact 6 o’clock position (Figure 6). Note how thin the joint capsule is here (even compared with the 5 o’clock view): at this level the capsule is no longer reinforced by the glenohumeral ligaments.

**Figure 7:**
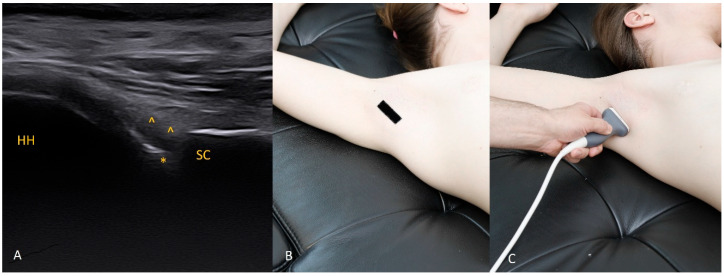
The 7 o’clock position. Relative to the 6 o’clock position (Figure 6), the transducer is shifted about 30° posteriorly; the outline of the scapular body disappears from the image.

**Figure 8:**
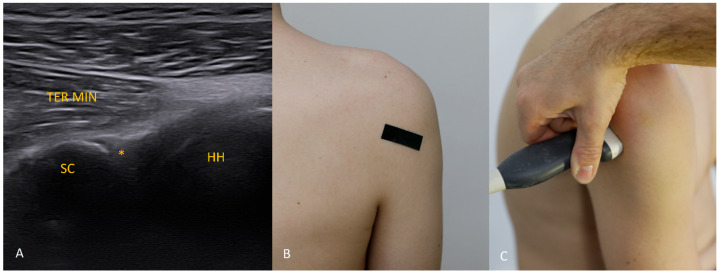
The 8 o’clock position. Starting from the 9 o’clock transducer position (see Figure 9), shift the transducer slightly inferiorly and rotate it about 30° caudally.

**Figure 9:**
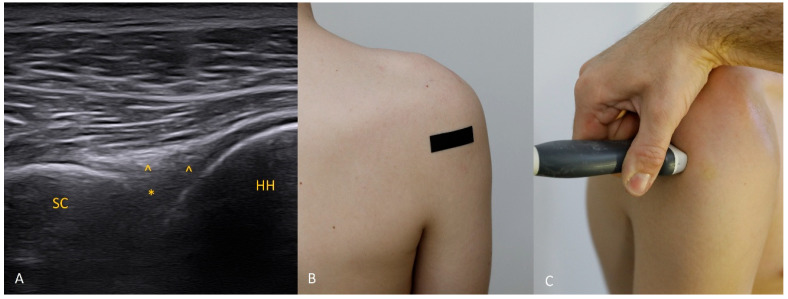
The 9 o’clock position. This view is generally the easiest to obtain. The transducer is aligned along the long axis of the scapula just below the scapular spine and held in a horizontal plane.

**Figure 10:**
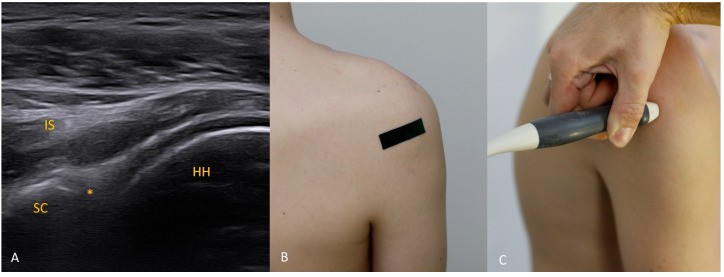
The 10 o’clock position. From the 9 o’clock image, shift the transducer slightly superiorly and rotate it about 30° cranially.

**Figure 11:**
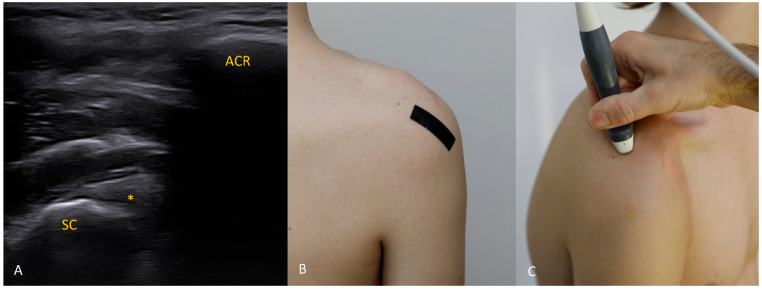
The 11 o’clock position. Assessment at 11 o’clock can be difficult because a portion of the labrum may lie in the acoustic shadow of the posterior acromial angle. Position the transducer so that the posterior acromial corner is seen on one side and the bony glenoid rim with the labrum on the other. If the labrum is obscured by acromial shadow, the issue can be resolved with trapezoidal imaging or by using a convex transducer.

**Figure 12:**
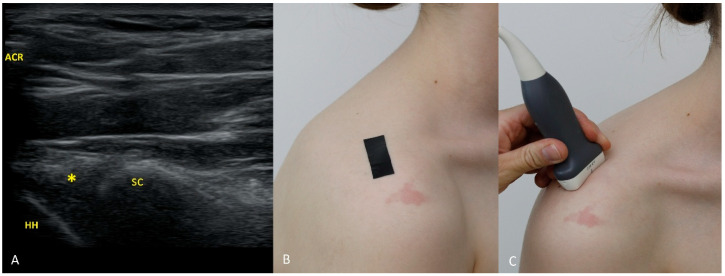
The 12 o’clock position. For the 12 o’clock view, place the transducer vertically over the supraspinatus fossa, in the soft spot anterior to the scapular spine, posterior to the clavicle, and medial to the acromion, with the transducer partly overlapping the medial acromial edge. Medial and deep to the acromion, the supraspinatus muscle is seen; deeper still, the outline of the scapula and the region of the suprascapular notch; more laterally, the bony glenoid rim. The labrum attaching to the glenoid is usually visible without additional maneuvers. Occasionally, however, the labrum lies in the acoustic shadow of the acromion, depending on minor anatomical variations. If the labrum is not visible after initial transducer placement, we recommend remaining with the linear transducer and activating trapezoidal imaging, or switching to a convex transducer. With trapezoidal imaging, ultrasound waves propagate not only perpendicularly to the transducer face but also at an angle (e.g., ~15°), which allows one to “peek” under the acromion. Note that in the central portion of the field the beams travel almost exclusively perpendicular to the transducer face; the benefit of the oblique beam occurs when the structure of interest (here, the labrum) is positioned near the edge of the field. A similar effect can be obtained with a convex transducer by placing the structure near the field margin. Using these techniques not only enables visualization of the superior labrum but often also of a portion of the long head of the biceps tendon.

Each figure (Figure 1, Figure 2, Figure 3, Figure 4, Figure 5, Figure 6, Figure 7, Figure 8, Figure 9, Figure 10, Figure 11 and Figure 12) illustrates the ultrasonographic appearance of the glenoid labrum at a given clock-face position. Panels: (A) ultrasound image; (B) diagram indicating transducer placement on the patient’s shoulder; (C) clinical photograph of transducer application. Symbols used: ✱ (asterisk), labrum; SC, scapula (bony margin of the glenoid); CR, coracoid process; HH, humeral head; ^, joint capsule; TER MIN, teres minor; IS, infraspinatus; ACR, acromion. Not all abbreviations appear in every figure.

The anterior labrum—particularly between the 2 and 4 o’clock positions—is among the most challenging portions to assess sonographically. This difficulty arises from the complex spatial orientation of the scapula, which is positioned obliquely relative to the chest wall at an angle of approximately 45°. Moreover, the glenoid surface itself is inclined relative to the long axis of the scapula, causing the anterior labrum to lie almost entirely within a sagittal plane. Because ultrasound structures are best evaluated when the beam is aligned with their long axis, accurate transducer alignment in this region requires specific patient and transducer adjustments.

The relationship between the scapula and the thoracic wall changes dynamically with arm rotation. Increasing external rotation reduces the scapular tilt, allowing the operator to orient the transducer more parallel to the scapular long axis and thereby approach the anterior glenoid margin more directly. Visualization can be further optimized by applying gentle, medially directed pressure with the inner edge of the transducer to improve acoustic coupling and minimize beam refraction at the glenoid contour.

When assessing the labrum at 5, 6, and 7 o’clock, we suggest examining the patient in the supine position with the arm maximally abducted and externally rotated. The examiner sits on the axillary side. We recommend starting at 6 o’clock and then moving the transducer about 30° anteriorly and posteriorly to display the labrum at 5 and 7 o’clock, respectively.

For the posterior portion, the patient is examined while seated. It is most comfortable for the examiner to sit behind the patient; alternatively, from the front, the examiner reaches posteriorly with the transducer. The arm is initially positioned in external rotation. We recommend starting at 9 o’clock and then proceeding to 8 and 10 o’clock.

**Figure 13:**
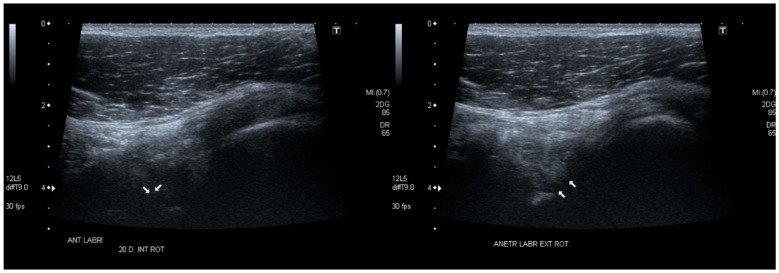
Anterior labral injury—assessment at the 3 o’clock position (Figure 3). Linear transducer with trapezoidal imaging (5–12 MHz). (**Left**): internal rotation of approximately 20°—the labrum is deformed and displaced medially. (**Right**): maximal external rotation—the labrum is reduced to its proper position under tension from the glenohumeral ligaments. Arrow, labrum.

**Figure 14:**
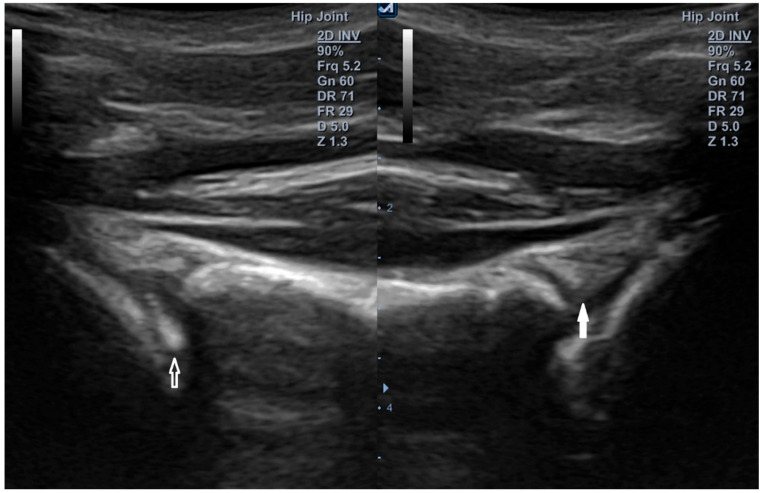
Superior labrum—12 o’clock position. Convex transducer (1–6 MHz). (**Left image**): superior labral tear (open arrow) of the right shoulder with displacement toward the joint interior. (**Right image**): intact superior labrum (white arrow) on the left side. Sixteen-year-old strength athlete.

Figure 13 and Figure 14: representative anterior (3–4 o’clock) and superior (12 o’clock) labral tears with dynamic displacement and contralateral normal comparison.

Pitfalls: acoustic shadow from acromion/coracoid (use trapezoidal/convex and edge-of-field placement), anisotropy at the labral margin, over-compression with the transducer masking subtle clefts [6,11,12,13,14,15,16,17].

Table 1 summarizes the standardized clock-face sonographic windows used for comprehensive glenoid labrum assessment. For each segment, the corresponding patient position, transducer type and orientation, recommended dynamic maneuvers, and common imaging pitfalls are listed to facilitate practical application of the protocol.

## 3. Results

A comprehensive 360-degree ultrasonographic assessment of the glenoid labrum was successfully completed in all 36 examined shoulders. The posterior (8–10 o’clock) and inferior (5–7 o’clock) segments consistently provided the highest image quality and the least operator-dependent variability. Visualization between 1 and 4 o’clock and between 11 and 12 o’clock occasionally required trapezoidal or convex imaging—most often due to acoustic shadowing from the coracoid process (1–2 o’clock) or acromion (11–12 o’clock), and the steep inclination of the anterior labrum relative to the ultrasound beam (3–4 o’clock).

In asymptomatic volunteers, the labrum appeared morphologically normal and continuous throughout its circumference. In symptomatic shoulders, the most common abnormalities included focal hypoechoic clefts, marginal irregularities, and partial detachment or flattening of the labral contour, most frequently at the anterior (3–4 o’clock) and superior (12 o’clock) positions. Dynamic maneuvers substantially improved diagnostic confidence by revealing motion-dependent findings—such as medial displacement of the anterior labrum during internal rotation (Bankart-type instability) and leaflet-like movement of the superior labrum at the biceps anchor (SLAP-type pathology).

All examinations were completed within a standardized protocol, and the procedure was well tolerated in all participants. Mild pain-related motion limitation occurred in a few symptomatic patients but did not preclude acquisition of diagnostic dynamic views. The results confirm the feasibility and reproducibility of the proposed clock-face ultrasonographic protocol, forming a solid basis for its further discussion and potential clinical implementation.

## 4. Discussion

In the broader context of shoulder instability work-up, magnetic resonance (MR) arthrography remains the reference standard for preoperative planning; however, real-world constraints—such as contraindications, limited access, and time-sensitive scenarios—frequently necessitate a first-line ultrasonographic pathway. Within this framework, our clock-face protocol functions as an adjunctive or alternative triage tool, delivering dynamic information that is directly actionable at the point of care [18,19,20,21,22].

This standardized protocol bridges the conceptual gap between arthroscopic anatomy and ultrasound imaging, providing a shared descriptive framework for both radiologists and orthopedic surgeons. By translating the arthroscopic clock-face orientation into a reproducible sonographic map, the method facilitates precise correlation of imaging findings with surgical anatomy. Such standardization may improve interdisciplinary communication and education, particularly for less experienced operators who rely on consistent spatial reference points.

The protocol proved capable of depicting the entire glenoid rim in most shoulders, enabling comprehensive evaluation of the labrum’s morphology and dynamic behavior. In contrast to conventional static imaging, the dynamic component of the examination allows real-time assessment of labral motion, marginal irregularities, and subtle detachments under stress maneuvers. These features are of particular clinical value in identifying early or partial labral pathology that might otherwise remain undetected on routine MR imaging. Importantly, this technique does not aim to replace MR arthrography but rather to complement it, especially in situations where MRI is contraindicated or impractical. Its portability and immediacy make it especially suited for sports medicine, emergency settings, and postoperative follow-up, where rapid, bedside assessment of shoulder stability is required [23,24,25,26,27,28,29,30,31,32,33,34,35,36].

As with all sonographic techniques, operator experience and technical training significantly influence diagnostic performance. The proposed protocol mitigates this limitation by providing clearly defined anatomical landmarks, transducer orientations, and dynamic maneuvers for each clock-face position. Structured implementation in musculoskeletal ultrasound curricula could further enhance inter-operator reproducibility and foster consistency across institutions. Moreover, the development of standardized documentation—such as summary tables or diagrammatic reporting templates—may facilitate both clinical communication and future research on diagnostic accuracy [37,38,39,40,41].

Previous attempts to visualize the glenoid labrum with ultrasound have been reported, notably by Krzyżanowski and Park, who described early efforts to delineate labral anatomy and pathology sonographically [42,43]. However, these studies were limited in scope and did not establish a reproducible, position-by-position framework. The present work expands on their foundational observations, introducing a precise, systematic, and 360-degree protocol that aligns ultrasound interpretation with arthroscopic orientation and provides a unified platform for further validation and training.

## 5. Limitations

This study was primarily technical and methodological in nature and did not include a diagnostic accuracy comparison with MR arthrography or arthroscopy. The number of examined cases was limited, and the observed pathologies did not represent the full clinical spectrum. Ultrasound assessment of the glenoid labrum is also highly operator-dependent, requiring precise transducer handling and awareness of anisotropy artifacts. Additional challenges may arise from restricted shoulder mobility or the need to use trapezoidal or convex imaging modes to visualize the superior labral segments [44,45].

## 6. Conclusions

A structured 360-degree clock-face protocol enables comprehensive ultrasonographic assessment of the glenoid labrum through both static and dynamic maneuvers. By optimizing transducer orientation and applying trapezoidal or convex imaging when necessary, all labral quadrants can be consistently visualized. The proposed technique is particularly valuable when MR or MR arthrography is contraindicated or unavailable, serving as a practical first-line or complementary diagnostic tool in sports medicine, outpatient orthopedics, and postoperative follow-up. Future prospective studies should evaluate segment-specific diagnostic accuracy and reproducibility, define learning curves, and determine how protocol-based ultrasound may best complement MRI/MRA or arthroscopy in clinical decision-making.

## Figures and Tables

**Table 1:** Summary of the clock-face ultrasonographic protocol for glenoid labrum assessment.

Clock-Face Position	Patient Position	Transducer Orientation	Recommended Dynamic Maneuver	Key Anatomical Landmarks/Pitfalls
1–2 o’clock (anterosuperior)	Seated, arm in slight external rotation	Oblique coronal plane along anterior acromial margin	Gentle abduction and external rotation	May visualize sublabral foramen—do not misinterpret as detachment
3–4 o’clock (anterior)	Seated or supine	Longitudinal to anterior glenoid rim, medial transducer pressure	External–internal rotation	Evaluate the resting position of the labrum and its displacement pattern during rotational maneuvers
5–7 o’clock (inferior)	Supine with maximal abduction and external rotation	In a plane parallel to the plane of the scapular body approached from the axilla	External–internal rotation	Start at 6 o’clock and rotate ~30° anteriorly/posteriorly to display 5 and 7 o’clock
8–10 o’clock (posterior)	Seated, starting position—internal rotation	Horizontally, just below the scapular spine.	External–internal rotation	The 9 o’clock view is the simplest; rotate slightly caudally/cranially for 8 and 10 o’clock.
11 o’clock (posterosuperior)	Seated, arm neutral	Oblique coronal plane below acromial angle	External–internal rotation and elevation	Use trapezoidal mode or a convex transducer to ‘peek’ under the acromion.
12 o’clock (superior)	Seated, arm neutral	Coronal plane at the “soft spot” medial to the acromion	Rotational movements combined with adduction and abduction	Biceps–labral complex; beware of sublabral recess mimicking a tear; use trapezoidal mode or a convex transducer if necessary

Footnotes: The full scanning sequence requires approximately 5–8 min in a cooperative patient. The examination may be performed with a linear transducer, with trapezoidal imaging mode activated when additional field of view is required; if acoustic shadowing persists, a convex transducer can be employed. Dynamic maneuvers should be applied because certain types of lesions may become apparent only during motion.

## Data Availability

No new data were created or analyzed in this study.
